# RK3, a G-Type LecRLK, Interacts with FLS2 and BAK1 to Promote flg22-Triggered Immunity

**DOI:** 10.3390/biology15110822

**Published:** 2026-05-23

**Authors:** Lu Zhang, Zhengdong Yuan, Lingya Yao, Hui Xiao

**Affiliations:** 1 School of Environmental and Chemical Engineering, Shanghai University, Shanghai 200444, China; 2 Shanghai Key Laboratory of Plant Molecular Sciences, College of Life Sciences, Shanghai Normal University, Shanghai 200234, China

**Keywords:** G-type LecRLK, pattern-triggered immunity, flg22 signaling, flagellin-induced receptor heteromer, plant disease resistance

## Abstract

Lectin receptor-like kinases (LecRLKs) are a large subfamily of receptor-like kinases (RLKs), and their N-terminal lectin domain is predicted to reversibly bind to carbohydrates. Within this family, G-type LecRLKs represent a distinct subclass defined by an extracellular S-locus glycoprotein (SLG) domain, which was originally identified for its role in governing self-incompatibility in *Brassica* species. Emerging evidence suggests that G-type LecRLKs are involved in plant immunity; however, only a small fraction have been functionally characterized, leaving the roles of most family members largely unknown. In this study, we identified *RK3* (Receptor Kinase 3) as the most strongly induced gene within the G-type LecRLK clade VI upon infection with *Pseudomonas syringae* pv. *tomato* DC3000 (*Pst* DC3000). Through both gain- and loss-of-function analyses, we demonstrated that RK3 positively regulates flg22-induced immune signaling events, including oxidative burst and mitogen-activated protein kinase (MAPK) activation, as well as downstream responses such as defense gene expression and ethylene production. Remarkably, the immune-enhancing activity of RK3 does not require its kinase domain. Critically, both full-length RK3 and a kinase-deleted variant (RK3-ΔK) constitutively interact with FLS2 (Flagellin-Sensing 2) and BAK1 (BRASSINOSTEROID INSENSITIVE 1-associated receptor kinase 1). This provides direct evidence that RK3 functions primarily as a co-regulatory component within the PRR complex, independent of its kinase activity. Moreover, ectopic expression of RK3 in tomato enhanced resistance to *Pst* DC3000, highlighting its potential utility in engineering disease resistance in crops. Thus, RK3 reveals a non-canonical, kinase-independent mechanism by which a G-type LecRLK potentiates plant immunity, expanding our understanding of RLK signaling complexity.

## Simple Summary

Plants are constantly threatened by disease-causing bacteria. To defend themselves, they rely on sensors on their cell surfaces that detect invading germs and turn on immune responses. In this study, we asked which plant genes are switched on quickly during infection and whether any of them could be used to make crops more resistant. We discovered a gene called *Receptor Kinase 3* (or *RK3* for short) in the model plant *Arabidopsis*. When this gene is active, it helps the plant mount a stronger defense against bacteria. Surprisingly, RK3 works in an unusual way: it does not need the typical “kinase” activity that most similar defense proteins require. Instead, it simply attaches to the main immune sensor complex and boosts its signaling. Importantly, when we put the same *RK3* gene into tomato plants, they also became more resistant to bacterial infection. Our findings reveal a new trick that plants use to fight diseases, and they suggest that *RK3* could be a useful tool for breeding or engineering crops with better disease resistance, helping to reduce the use of chemical pesticides.

## 1. Introduction

Plants possess a sophisticated, multi-layered innate immune system that primarily comprises two interconnected defense tiers: pattern-triggered immunity (PTI) and effector-triggered immunity (ETI) [1,2]. PTI is initiated when cell-surface pattern recognition receptors (PRRs) detect conserved microbe- or pathogen-associated molecular patterns (MAMPs or PAMPs), thereby establishing basal defense responses [3,4]. To counteract this, pathogens secrete effector proteins that suppress host immune signaling. In turn, these effectors can be recognized by plant intracellular resistance proteins, leading to activation of ETI, which is often associated with a hypersensitive response and programmed cell death that effectively restrict pathogen spread [5,6].

As the first line of defense in plant immunity, PRR-mediated recognition plays a central role in immune activation. All known PRRs are cell-surface localized receptor-like kinases (RLKs) or receptor-like proteins (RLPs), both of which contain an extracellular domain (ECD) and a single-pass transmembrane helix. The primary structural distinction is that RLKs possess an intracellular kinase domain, whereas RLPs lack this catalytic activity. These receptor families have undergone extensive expansion in plant genomes, and constitute a substantial proportion of protein-coding genes [7]. For instance, the *Arabidopsis* genome encodes more than 600 RLKs, representing approximately 2.5% of all protein-coding genes, underscoring their broad involvement in diverse biological processes, including immune signaling [8,9,10].

Emerging evidence has extended the functional scope of plant cell-surface receptors from pathogen recognition to the perception of abiotic environmental cues, particularly temperature-related stress [11]. For instance, heat stress compromises the accumulation of the prototypical PRR FLS2 and attenuates flg22-induced immune outputs, whereas cold stress transcriptionally upregulates several PRRs, including FLS2 and its co-receptor BAK1. Moreover, certain receptors, such as the leucine-rich repeat (LRR) RLK KOIN (KINASE ON THE INSIDE), directly participate in cold signaling [12]. Together, these findings highlight the necessity of characterizing PRR functions under complex, real-world conditions where biotic and abiotic stresses frequently co-occur.

Most well-characterized PRRs contain leucine-rich repeat (LRR) ECDs, which represent the most prevalent class of plant RLKs and RLPs. One of the best-studied LRR-RLKs in *Arabidopsis* is FLS2, which recognizes the bacterial peptide epitope flg22 derived from flagellin. Upon flg22 perception, the co-receptor BAK1 associates with FLS2 and phosphorylates *Botrytis*-induced kinase 1 (BIK1), thereby initiating downstream signaling cascades that promote plant immune responses [13].

Although most characterized PRRs possess an LRR-type ECD, several additional receptor families have also been identified in plants. The *Arabidopsis* genome encodes approximately 75 lectin receptor-like kinases (LecRLKs), including 42 L-type, 32 G-type, and 1 C-type members [14,15]. L-type LecRLKs harbor a single legume-lectin domain in their extracellular region, and C-type LecRLKs contain a calcium-dependent lectin domain, whereas G-type LecRLKs contain an α-D-mannose-binding bulb lectin domain together with an S-locus glycoprotein (SLG) domain, and may additionally contain an epidermal growth factor (EGF) domain and/or a plasminogen-apple-nematode (PAN) domain. For example, RK3 contains a PAN domain. Notably, LecRLKs appear to be plant-specific, as no homologs have been identified in yeast or human genomes. Emerging evidence indicates that LecRLKs play important roles in plant innate immunity [16]. Initially predicted to bind carbohydrate-associated ligands due to their lectin domains, several LecRLKs have since been shown to function as receptors for extracellular ATP (eATP), NAD^+^ (eNAD^+^), NADP^+^ (eNADP^+^), and the bacterial metabolite 3-OH-C10:0, mediating plant immune responses [15]. Although many *LecRLK*s are transcriptionally induced during pathogen invasion or tissue damage [17,18], the biological functions and molecular mechanisms of most LecRLKs in plant immunity remain poorly understood.

Using gene expression analysis from Expression Atlas (https://www.ebi.ac.uk/gxa, accessed on 27 January 2026), we identified *RK3* (*AT4G21380*) as one of the most strongly induced G-type LecRLK genes following treatment with flg22. A previous study also reported that *RK3* expression is upregulated by the intracellular coiled-coil nucleotide-binding leucine-rich repeat (CC-NB-LRR) receptor proteins RPP7 and RPP4 in response to the oomycete pathogen *Peronospora parasitica*, suggesting a potential role in *Arabidopsis* immunity [19]. However, the molecular mechanisms of *RK3* in plant immunity remain unclear.

In this study, we demonstrated that *RK3* expression is strongly induced following infection with *Pseudomonas syringae* pv. *tomato* DC3000 (*Pst* DC3000). Further genetic analyses using *RK3* overexpressing plants and T-DNA insertion mutants demonstrated that RK3 positively regulates flg22-triggered immune responses, including MAPK activation, reactive oxygen species (ROS) burst, defense gene expression, and resistance to *Pst* DC3000. Co-immunoprecipitation assays revealed that RK3 constitutively interacts with the PRR complex components FLS2 and BAK1. Notably, a kinase domain-deleted version of RK3 (RK3-ΔK) maintained its interaction with FLS2 and BAK1, and retained immune-enhancing activity, suggesting that the extracellular domain of RK3 through its simultaneous association with FLS2 and BAK1, supports flg22 signaling without requiring kinase activity. Furthermore, interfamily transfer of *RK3* into tomato significantly enhanced disease resistance, highlighting its potential utility for engineering disease resistance in crops. Collectively, our findings identified RK3 as a novel positive regulator of pattern-triggered immunity and revealed a previously unrecognized functional mode of action for G-type LecRLKs.

## 2. Materials and Methods

### 2.1. Biological Materials

Wild-type controls consisted of *Arabidopsis thaliana* (Col-0) and tomato (*Solanum lycopersicum* cv. Micro-Tom). The T-DNA insertional lines *rk3* (SALK_109125) and *rk3-2* (SALK_001986) were obtained from the *Arabidopsis* Biological Resource Center (ABRC).

For transcriptional profiling, MAPK activity assays, and ethylene production measurements, surface-sterilized seeds were plated onto half-strength Murashige and Skoog (MS) medium and kept at 22 °C under continuous illumination (60 μE m^−2^ s^−1^) for 7 days. They were then transferred into sealed gas chromatography (GC) vials containing liquid half-strength MS medium and incubated for an additional 3–10 days. Thereafter, the seedlings were treated with 10 μM estradiol, 100 nM flg22, 100 nM/300 nM/1 μM flgII-28, or the corresponding solvent controls. Ethylene accumulation was recorded at predetermined time points, and tissue samples were harvested for reverse transcription quantitative PCR (RT-qPCR) or immunoblotting.

For protoplast isolation, ROS burst measurements, and pathogen challenge experiments, plants were grown in soil under a 14 h light/10 h dark cycle at 22 °C for 4 weeks preceding leaf collection.

### 2.2. Molecular Cloning and Plant Transformation

The coding sequence (CDS) of *RK3* was retrieved from the TAIR database (https://www.arabidopsis.org/, accessed on 6 October 2021). Primers were designed using SnapGene software (version 6.0, from GSL Biotech LLC, Chicago, IL, USA): the forward primer incorporated *Sal*I and *Bam*HI sites, and the reverse primer incorporated *Spe*I and *Stu*I sites (all restriction enzymes were purchased from Thermo Fisher Scientific, Waltham, MA, USA). Primers were synthesized by Sangon Biotech (Shanghai, China). The CDS was amplified from Col-0 cDNA by PCR using FastPfu high-fidelity DNA polymerase (TransGen Biotech, Beijing, China) and first ligated into a *pMD19T-vector* (Takara, Kusatsu, Japan). After Sanger sequencing verification (Sangon Biotech), the confirmed fragment was digested with *Sal*I and *Spe*I and ligated into the estradiol-inducible *pER8-Est:HA* vector to generate *Est:RK3-HA*. For transient protoplast assays, the same verified CDS was digested with *Bam*HI and *Stu*I and inserted into *pHBT-35S:FLAG*, yielding *35S:RK3-FLAG*. All constructs were verified by colony PCR and restriction digestion before use.

The kinase-domain-deleted variant, RK3-ΔK, was generated by designing a reverse primer immediately upstream of the predicted kinase domain (deleting the kinase domain and all subsequent sequences), while retaining the same forward and reverse restriction sites (*Sal*I/*Bam*HI and *Spe*I/*Stu*I, respectively) as for the full-length RK3. Using the sequence-verified *pMD19T-RK3* as template, the truncated CDS was amplified by PCR and then cloned into *pER8-Est:HA* and *pHBT-35S:Myc* following the same procedure (*pMD19T-vector* cloning, Sanger sequencing, restriction digestion with *Sal*I/*Spe*I for *pER8* and *Bam*HI/*Stu*I for *pHBT*), resulting in *Est:RK3-ΔK-HA* and *35S:RK3-ΔK-Myc*, respectively.

For co-immunoprecipitation experiments, the CDSs of FLS2 and BAK1 were individually amplified and cloned into *pHBT-35S:HA* via *Bam*HI and *Stu*I sites, yielding *35S:FLS2-HA* and *35S:BAK1-HA*. The same *pMD19T-vector* and sequencing steps were applied to ensure sequence fidelity. All primer sequences used for these constructs are listed in Supplementary Table S2.

*Arabidopsis* transformants (Col-0 background) were generated via *Agrobacterium*-mediated floral dip [20]. For each construct, roughly 50 T1 lines were examined for transgene expression by anti-HA immunoblotting (anti-HA antibody from Thermo Fisher Scientific). Among these, T2 lines harboring a single T-DNA insertion (confirmed by segregation analysis on hygromycin-containing medium) and displaying robust, reproducible transgene expression were selected for all subsequent assays. To conserve space in the main figures, data from one representative line are presented; this line faithfully reflected the phenotypes observed across multiple independent transformants. All results from another independent line are shown in the Supplementary Figures.

### 2.3. Bioinformatic Analysis

Protein sequences of RK1, RK2, and RK3 were downloaded from the TAIR database. A maximum-likelihood phylogenetic tree was generated using MEGA (version 11.0.13), and conserved domains were annotated based on the NCBI Conserved Domain Database (https://www.ncbi.nlm.nih.gov/cdd/, accessed on 15 August 2024). The domain architecture of RK3 was additionally predicted with the online tools SMART (https://smart.embl.de/smart/change_mode.cgi, accessed on 5 January 2025) and TMHMM (http://www.cbs.dtu.dk/services/TMHMM/, accessed on 5 January 2025).

### 2.4. Transcript Level Analysis

RNA extraction and cDNA synthesis were performed according to the manufacturer’s instructions (TRIzol, Invitrogen, Carlsbad, CA, USA; PrimeScript RT, Takara). Relative transcript levels were determined by RT-qPCR using a CFX Connect Real-Time PCR System (Bio-Rad, Hercules, CA, USA) with SYBR Green detection (Toyobo, Osaka, Japan), as described previously [21]. *EF1α* served as the endogenous control for *Arabidopsis*, and *ACT* for tomato. Three biological replicates were performed for each genotype and treatment. Relative expression was calculated using the 2−ΔΔCt method [22] and presented either as fold change relative to untreated controls or as a percentage of the reference gene transcript level. All primer sequences are listed in Supplementary Table S1.

### 2.5. Ethylene Quantification

Ethylene production was quantified by gas chromatography (PANNA A60, Beijing, China) as described previously [21]. Two-week-old *Arabidopsis* or 2.5-week-old tomato seedlings grown in GC vials with hydroponic medium were treated with 10 μM estradiol (Est), 100 nM flg22 (for *Arabidopsis*), 100 nM flgII-28 (for tomato), or solvent controls (0.1% DMSO for estradiol). A standard curve was generated using ethylene standard gas (99.9% purity, National Institute of Metrology, Beijing, China). At specified times post-elicitation, headspace gas was collected, and ethylene concentrations—normalized to fresh weight—were derived from standard curves. Three independent biological replicates (10 *Arabidopsis* or 3 tomato seedlings per vial) were analyzed for each treatment and time point.

### 2.6. Co-Immunoprecipitation and Immunoblotting Assays

Protoplast isolation, transformation, and co-immunoprecipitation (co-IP) assays were carried out as described previously [21] with the following buffer specifications. The washing buffer contained 100 mM NaCl, 10 mM HEPES (pH 7.5), 1 mM EDTA (pH 8.0), 10% (*v*/*v*) glycerol, and 0.5% (*v*/*v*) Triton X-100. The lysis buffer was prepared by adding the following inhibitors per 1 mL of washing buffer immediately before use: 2 μL of 1 M NaF (final 2 mM), 2 μL of 1 M Na_3_VO_4_ (final 2 mM), 2.5 μL of 0.4 M DTT (final 1 mM), and 20 μL of 50× protease inhibitor cocktail (Thermo Fisher Scientific; final 1×). After an overnight recovery period, the transformed protoplasts were exposed to 100 nM flg22 for 10 min prior to protein extraction. Immunoprecipitation was conducted using anti-FLAG (Thermo Fisher Scientific), anti-BAK1 (Agrisera, Vännäs, Sweden), or anti-Myc (Sigma-Aldrich, St. Louis, MO, USA) antibodies, and subsequent immunoblotting was performed with anti-HA, anti-FLAG, anti-Myc, anti-FLS2 (Agrisera), or anti-BAK1 antibodies, all following published procedures [21].

For MAPK activation assays, total proteins extracted from 10-day-old *Arabidopsis* or 14-day-old tomato seedlings treated with 100 nM flg22 or 300 nM flgII-28 (sampled at the indicated time points). The extraction buffer was the same as the lysis buffer described above. Protein extracts were subjected to immunoblot analysis using anti-phospho-ERK1/2 antibody (Sigma-Aldrich) as described previously [21].

### 2.7. Measurement of Reactive Oxygen Species Burst

To measure ROS production, a luminol-based chemiluminescence assay was employed as described previously [23]. Leaf discs (5 mm diameter) excised from 4-week-old plants were pre-incubated in either sterile water or 10 μM estradiol for 24 h. After this treatment, the discs were transferred into a reaction mixture containing 100 nM flg22, 100 μM luminol (Sigma-Aldrich), and 10 μg/mL horseradish peroxidase (HRP, Sigma-Aldrich). Luminescence was then recorded at 2 min intervals for a total of 60 min using a GloMax microplate luminometer (Promega, Madison, WI, USA). For each treatment, at least 20 leaf discs were used as biological replicates, and the data are presented either as kinetic curves of relative luminescence over time or as integrated relative light units (RLU).

### 2.8. Evaluation of Pathogen Resistance

For disease resistance assays, leaves of 4-week-old transgenic plants were first infiltrated with either 10 μM estradiol or the corresponding solvent control (0.1% DMSO). After 24 h, the same leaves were challenged with a bacterial suspension of *Pst* DC3000 adjusted to an optical density at 600 nm (OD_600_) of 0.0005 in 10 mM MgCl_2_. Three days post-inoculation, bacterial multiplication was determined as described previously [21]. In brief, leaf discs (0.2 cm^2^ each) were collected from three individual plants per genotype and treatment (serving as three biological replicates), pooled, homogenized, serially diluted, and plated on Luria–Bertani (LB) agar containing 50 μg/mL rifampicin (Yeasen Biotech, Shanghai, China). Each dilution was plated in triplicate as technical replicates.

### 2.9. Generation of Transgenic Tomato Plants

Tomato (*Solanum lycopersicum* cv. Micro-Tom) transformation was carried out as described previously, with modifications [24]. Leaf explants (5 mm × 5 mm) excised from 10-day-old seedlings were preincubated for 1 day on T1 medium (MS salts containing 3% sucrose, 2 mg/L zeatin, and 0.1 mg/L IAA). The explants were then infected with an *Agrobacterium tumefaciens* strain GV3101 suspension (OD_600_ = 0.3 in T1 medium supplemented with 100 μM acetosyringone, Sigma-Aldrich) for 10 min, followed by co-cultivation on the same medium for 2 days in the dark. After co-cultivation, explants were moved to selection medium (T1 medium with 10 mg/L hygromycin and 200 mg/L timentin) to induce shoot regeneration. Regenerated shoots of 2–3 cm in length were excised and rooted on half-strength MS medium containing 3% sucrose, 2 mg/L IBA, and 100 mg/L timentin. Putative transgenic lines were initially screened by hygromycin resistance, and positive transformants were confirmed by immunoblotting using anti-HA antibody. For each construct, at least two independent T1 lines were selected and propagated to the T2 generation; all subsequent experiments were performed using T2 plants derived from these independent lines.

### 2.10. Data Analysis and Statistics

Results are shown as mean ± standard deviation (SD), with the number of independent biological replicates (*n* ≥ 3) indicated in each figure legend. Pairwise comparisons between two groups were evaluated using two-tailed Student’s *t*-test. For multiple comparisons, one-way or two-way analysis of variance (ANOVA) followed by Tukey’s honest significant difference (HSD) post hoc test was applied. Statistical analyses were performed using commercial software (IBM SPSS Statistics version 27, IBM, Armonk, NY, USA; GraphPad Prism version 9.5.1, GraphPad Software, San Diego, CA, USA). Significance levels are denoted as follows: * *p* < 0.05, ** *p* < 0.01, *** *p* < 0.001 for pairwise tests, and different lowercase letters (*p* < 0.05) for multiple comparisons.

## 3. Results

### 3.1. RK3 Expression Shows the Strongest Induction Among G-Type LecRLK Clade VI Members in Response to Pathogen Treatment

**Figure 1:**
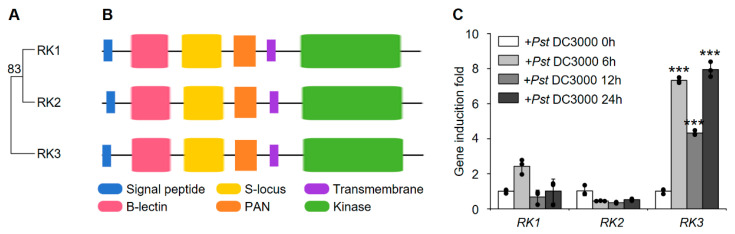
In silico and expression profiling of *RK3* and its two close homologs. (**A**) Phylogenetic relationships of RK1, RK2 and RK3. A maximum-likelihood (ML) tree was built using the full-length amino acid sequences of RK3 and its two most closely related paralogs. Bootstrap values (5000 replicates) are indicated at branching nodes. (**B**) Conserved domain organization of RK1, RK2 and RK3. The extracellular B-lectin, S-locus glycoprotein (SLG) and PAN domains, together with the transmembrane (TM) and intracellular protein kinase (PK) domains, are shown schematically. Domain predictions were performed using SMART and Pfam. (**C**) Transcriptional activation of *RK3* upon *Pseudomonas syringae* pv. *tomato* DC3000 (*Pst* DC3000) challenge. Ten-day-old *Arabidopsis* (Col-0) seedlings were inoculated with *Pst* DC3000 at OD_600_ = 0.02. Total RNA was extracted at the indicated time points and subjected to RT-qPCR. Transcript levels of *RK3* were normalized to the internal reference gene *EF1α* and are presented as fold change relative to the 0 h time point (set to 1). Data represent means ± SD from three biological replicates, with individual data (black dots) overlaid. Statistical comparisons against the 0 h control were performed using two-tailed Student’s *t*-test. ***, *p* < 0.001.

Using gene expression analysis from Expression Atlas (https://www.ebi.ac.uk/gxa, accessed on 27 January 2026), we identified *RK3* (*AT4G21380*) as one of the most strongly induced G-type LecRLK genes following treatment with flg22 (Figure S1). Using the RK3 protein sequence as a query in a Basic Local Alignment Search Tool (BLAST 2.9.0+) search against the TAIR database, we identified two closely related orthologs, *RK1* (*AT1G65790*) and *RK2* (*AT1G65800*), which share 74% and 75% sequence identity with the protein sequence of *RK3*, respectively (Figure 1A). Conserved domain analysis revealed that all three proteins contain a B-lectin domain, an SLG domain, and a PAN domain within their extracellular regions (Figure 1B), forming the clade VI A1b in *Arabidopsis* G-type LecRLKs [25].

To compare the expression dynamics of these three homologs during pathogen invasion, we performed a reverse transcription-quantitative polymerase chain reaction (RT-qPCR) analysis following a time-course analysis of *Pst* DC3000 inoculation. Among the three genes, *RK3* expression exhibited the most rapid and pronounced induction, with transcript levels markedly increasing eight-fold at 6 h post-inoculation with *Pst* DC3000 (Figure 1C). Further expression profiling following infection with *Pst* DC3000 strains carrying AvrRpt2 or AvrRpm1, as well as the fungal pathogen *Botrytis cinerea*, consistently demonstrated strong upregulation of *RK3* expression (Figure S2). Collectively, these results suggest that *RK3* may function as a prominent early-responsive regulator in disease resistance in *Arabidopsis*.

### 3.2. RK3 Potentiates flg22-Triggered Immune Signaling

**Figure 2:**
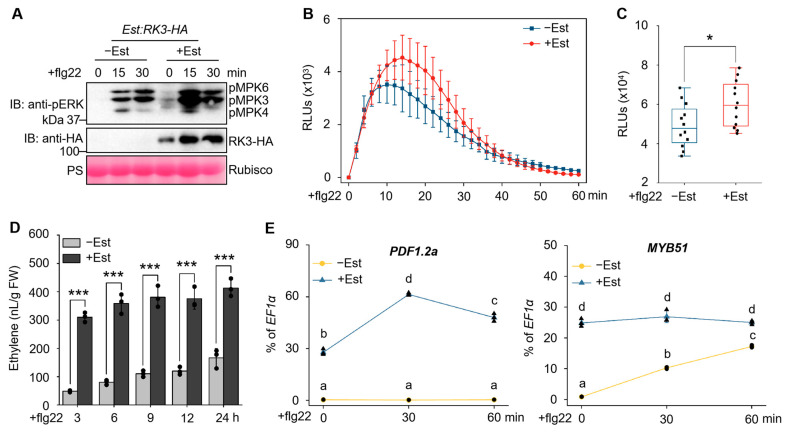
RK3 positively modulates flg22-induced immune responses in *Arabidopsis*. *Est:RK3-HA* transgenic seedlings were pre-treated with 10 µM estradiol (+Est) or solvent control (−Est) for 24 h and then exposed to 100 nM flg22. Plant materials included aseptically grown seedlings (10-day-old for (**A**,**E**); 14-day-old for (**D**)) and soil-grown rosette leaves (4-week-old for (**B**,**C**)). (**A**) Enhanced MAPK activation by RK3 upon flg22 treatment. Immunoblots employed anti-pERK (for phosphorylated MPK3/6) and anti-HA (for RK3) antibodies. Rubisco staining served as a loading control. (**B**,**C**) RK3 amplifies the flg22-triggered oxidative burst. ROS production was monitored over time using a luminol-based assay. (**B**) Kinetic curves are means ± SD (*n* = 12 leaf discs). RLU, relative light units. (**C**) Box plot shows integrated ROS levels (total photon counts within 0–60 min). Significance was assessed by Student’s *t*-test (*, *p* < 0.05; *n* = 12). (**D**) RK3 boosts flg22-induced ethylene emission. Ethylene accumulation was quantified by gas chromatography. Values are means ± SD of three independent biological replicates (10 seedlings each). FW, fresh weight. ***, *p* < 0.001 (Student’s *t*-test). (**E**) RK3 upregulates defense marker gene expression after flg22 challenge. Transcript levels of *PDF1.2a* and *MYB51* were determined by RT-qPCR and normalized to the internal control *EF1α*. Results are means ± SD (*n* = 3). Different lowercase letters above bars denote significant differences (two-way ANOVA with Tukey’s HSD post hoc test, *p* < 0.05). Black symbols in (**C**–**E**) represent individual data points.

As RK3 is a potential early-responsive regulator, it might contribute to PTI. We therefore investigated whether *RK3* overexpression could enhance flg22-triggered immune responses. We generated estradiol (Est)-inducible *RK3*-overexpressing transgenic *Arabidopsis* plants (*Est:RK3-HA*) that do not exhibit altered growth phenotypes (Figure S11A), and in which *RK3* expression can be rapidly induced upon Est treatment. Seedlings were pretreated with Est to induce *RK3* expression and subsequently challenged with flg22 to assess immune responses. Pre-induction of *RK3* expression greatly enhanced flg22-induced activation of MPK3 and MPK6 (Figure 2A and Figure S3A). Similarly, *RK3* pre-induction significantly potentiated the flg22-triggered ROS burst (Figure 2B,C and Figure S3B,C). *RK3* overexpression also significantly increased ethylene production following flg22 treatment (Figure 2D and Figure S3D). Moreover, the flg22-induced expression of defense marker genes *PDF1.2a* and *MYB51* was substantially elevated by overexpression of *RK3* (Figure 2E and Figure S3E). Collectively, these data indicate that *RK3* overexpression promotes hallmark flg22-triggered immune responses.

**Figure 3:**
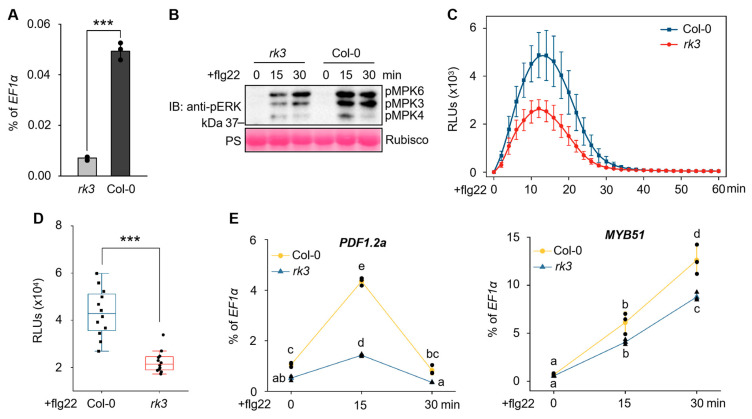
Loss of RK3 function compromises flg22-triggered immune responses in *Arabidopsis*. (**A**) Molecular verification of the *rk3* loss-of-function mutant (SALK_109125). Total RNA was extracted from 10-day-old seedlings, and *RK3* transcript abundance was measured by RT-qPCR with normalization to *EF1α*. Data are mean ± SD (*n* = 3). ***, *p* < 0.001 (Student’s *t*-test). (**B**) Impaired MAPK activation in the *rk3* mutant following flg22 elicitation. Seedlings were treated with 100 nM flg22 for the indicated times. Phosphorylation of MPK3/4/6 was detected by immunoblotting with anti-pERK antibodies. Rubisco staining served as a loading control. (**C**,**D**) Attenuated oxidative burst in *rk3* leaf discs after flg22 challenge. ROS production was monitored using a luminol-based assay. (**C**) Kinetics of ROS release (mean RLU ± SD, *n* = 12 leaf discs). RLU, relative light units. (**D**) Cumulative ROS levels (integrated photon counts from 0 to 60 min) are presented as a box plot (*n* = 12). Significance was determined by Student’s *t*-test (***, *p* < 0.001). (**E**) Reduced induction of defense-related genes in the *rk3* mutant upon flg22 treatment. Transcript levels of *PDF1.2a* and *MYB51* were quantified by RT-qPCR in 10-day-old seedlings treated with 100 nM flg22 for the indicated hours. Expression values were normalized to *EF1α*. Data are mean ± SD (*n* = 3 biological replicates). Different lowercase letters indicate statistically significant differences (two-way ANOVA with Tukey’s HSD post hoc test, *p* < 0.05). Black symbols in (**A**,**D**,**E**) represent individual data points.

To genetically validate the contribution of *RK3* in flg22-induced immunity, we obtained T-DNA insertional *rk3* (SALK_109125) and *rk3-2* (SALK_001986) mutants, both of which exhibited no detectable growth defects compared to wild-type plants (Figure S11B). RT-qPCR confirmed severely reduced *RK3* transcript abundance in the mutants, at 14.49% and 1.38% of the wild-type level (Figure 3A and Figure S4A). We then characterized flg22-triggered responses in T-DNA mutants plants. Immunoblot analysis revealed that flg22-activated phosphorylation of MPK3/6 was weaker in mutants (Figure 3B and Figure S4B). The amplitude of flg22-elicited ROS burst was also substantially reduced in mutants, though the duration of the ROS burst seemed similar to that of WT plants (Figure 3C,D and Figure S4C,D). Flg22-induced ethylene production was initially comparable but significantly reduced at later time points in the mutants compared to wild-type *Arabidopsis* (Figure S5). Consistently, flg22-induced upregulation of *PDF1.2a* was significantly reduced in mutants compared to WT at 0 min and 15 min, whereas *MYB51* upregulation was significantly reduced only at 30 min, with no significant difference at 0 min and 15 min (Figure 3E and Figure S4E). Collectively, these loss-of-function data demonstrate that *RK3* is necessary for sustaining the amplitude of early signaling and the full output of flg22-triggered immunity.

### 3.3. RK3 Constitutively Interacts with Both FLS2 and BAK1

**Figure 4:**
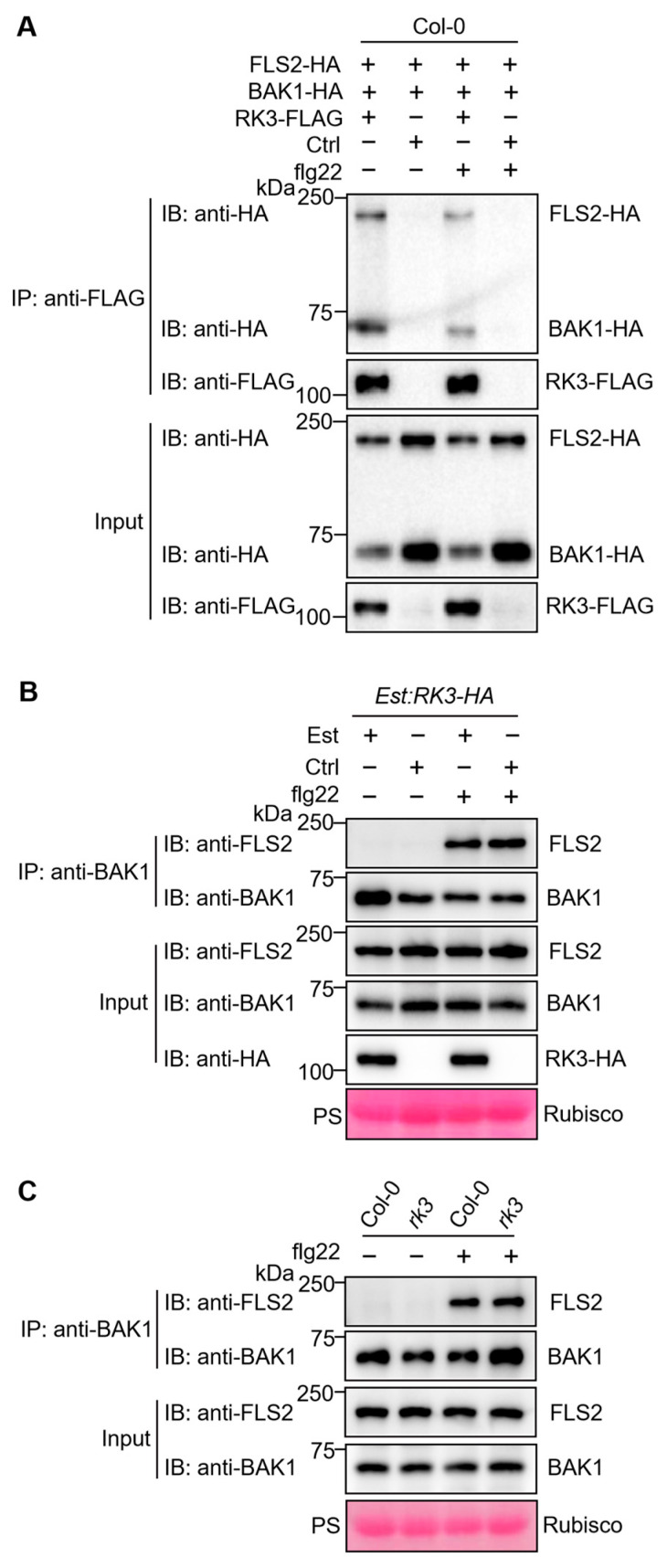
RK3 constitutively associates with FLS2 and BAK1 regardless of flg22 perception. (**A**) Ligand-independent interaction of RK3 with FLS2 and BAK1. RK3-FLAG was co-expressed with FLS2-HA or BAK1-HA in *Arabidopsis* protoplasts. After a 10 min exposure to 100 nM flg22 or mock control, protein complexes were immunoprecipitated using anti-FLAG beads and detected by immunoblotting with anti-FLAG and anti-HA antibodies. Ctrl, vector control. The faint band in the Ctrl lane of the anti-HA IP is due to sample spillover from the adjacent lane during loading and does not reflect genuine interaction. (**B**,**C**) RK3 is not required for flg22-triggered FLS2-BAK1 complex formation. Co-immunoprecipitation assays were performed using whole-seedling extracts. (**B**) Two-week-old *Est:RK3-HA* transgenic seedlings were pre-treated with 10 µM estradiol (+Est) or solvent control (−Est) for 24 h, followed by 100 nM flg22 for 15 min. (**C**) The *rk3* mutant and wild-type (Col-0) seedlings were treated under the same flg22 condition. Immunoprecipitates and input lysates were analyzed by immunoblotting with anti-BAK1, anti-FLS2 and anti-HA antibodies. Ctrl, mock treatment (−Est). In (**A**–**C**), “+” and “–” indicate the presence or absence of the indicated component (estradiol, flg22, or maxi-prep DNA).

To elucidate the molecular mechanism by which RK3 regulates flg22-triggered immunity, we first investigated its physical association with the core flg22 receptor complex-FLS2 and BAK1. Co-immunoprecipitation (co-IP) assays in *Arabidopsis* protoplasts showed that RK3 interacted with both FLS2 and BAK1 in the absence of flg22 (Figure 4A). Notably, flg22 treatment did not enhance the interaction between RK3 and FLS2/BAK1, indicating that RK3 associates with the FLS2-BAK1 complex constitutively.

We next tested whether RK3 could influence the flg22-induced formation of the FLS2-BAK1 complex. Using *Est:RK3-HA* overexpression plants and *rk3* mutants plants, together with antibodies of endogenous FLS2 and BAK1 for co-IP assays, we found that overexpression of *RK3* did not enhance the abundance of flg22-induced FLS2-BAK1 complexes, as determined by densitometric analysis of the co-immunoprecipitated bands normalized to the input loading controls (Figure 4B and Figure S6). Conversely, the loss of RK3 in *rk3* mutant also did not diminish the flg22-triggered association between FLS2 and BAK1 compared to that in WT plants (Figure 4C). Taken together, these results indicate that RK3 is constitutively associated with both FLS2 and BAK1, while it does not modulate flg22-induced receptor kinase complex assembly, distinguishing its mode of action from canonical scaffolding proteins in PTI signaling.

### 3.4. RK3-Mediated Potentiation of flg22-Triggered Immunity Is Independent of Its Kinase Activity

**Figure 5:**
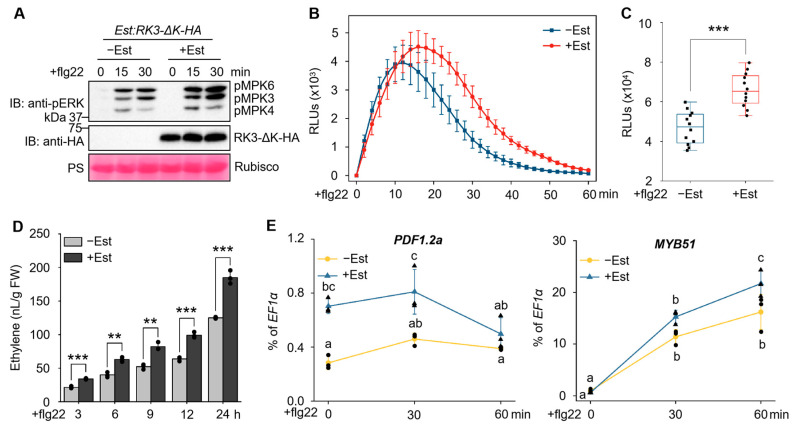
The kinase-deleted RK3 variant (RK3-ΔK) retains the ability to potentiate flg22-triggered immunity. *Est:RK3-ΔK-HA* transgenic seedlings were pre-treated with 10 µM estradiol (+Est) or solvent control (−Est) for 24 h, then challenged with 100 nM flg22. Plant materials included aseptically grown seedlings (10-day-old for (**A**,**E**); 14-day-old for (**D**)) or soil-grown rosette leaves (4-week-old for (**B**,**C**)). (**A**) RK3-ΔK expression enhances flg22-induced MAPK phosphorylation. Immunoblotting was performed with anti-pERK (for MPK3/4/6) and anti-HA (for RK3-ΔK) antibodies. Rubisco staining served as a loading control. (**B**,**C**) RK3-ΔK amplifies the flg22-triggered oxidative burst. ROS production was monitored over 60 min after flg22 application. (**B**) Kinetic curves are mean relative luminescence units (RLU) ± SD (*n* = 12 leaf discs). (**C**) Box plot shows integrated ROS levels (total photon counts, 0–60 min). Significance was assessed by Student’s *t*-test (***, *p* < 0.001; *n* = 12). (**D**) RK3-ΔK boosts flg22-induced ethylene emission. Ethylene accumulation was quantified by gas chromatography in 14-day-old seedlings at the indicated hours after elicitation. Data are mean ± SD (*n* = 3). Student’s *t*-test: **, *p* < 0.01; ***, *p* < 0.001. (**E**) RK3-ΔK upregulates defense marker genes upon flg22 treatment. Transcript levels of *PDF1.2a* and *MYB51* were measured by RT-qPCR in 10-day-old seedlings (100 nM flg22, the indicated hours). Expression values were normalized to *EF1α*. Data are mean ± SD (*n* = 3 biological replicates). Different lowercase letters denote significant differences (two-way ANOVA with Tukey’s HSD post hoc test, *p* < 0.05). Black symbols in (**C**–**E**) represent individual data points.

Receptor-like kinases (RLKs) typically transduce signals through ligand-induced activation of their intracellular kinase domains. To determine whether the kinase activity of RK3 is required for its function in immunity, we generated estradiol-inducible transgenic plants expressing a kinase-deleted version of RK3 (*Est:RK3-ΔK-HA,* Figure S7), which exhibited no detectable effect on growth phenotypes (Figure S11C). Similar to what we observed in *Est:RK3-HA* plants, overexpression of *RK3-ΔK* significantly enhanced multiple flg22-triggered immune responses. Immunoblot analysis showed that *RK3-ΔK* overexpression potentiated the activation of MPK3/6 following flg22 treatment (Figure 5A and Figure S8A). Furthermore, *RK3-ΔK* overexpression enhanced the flg22-induced oxidative burst, resulting in both higher peak intensity and greater total accumulation of ROS (Figure 5B,C and Figure S8B,C). Consistent with these early and mid-phase responses, flg22-elicited ethylene production was also markedly increased in *RK3-ΔK* overexpression plants (Figure 5D and Figure S8D). Moreover, transcript levels of the defense-related genes *PDF1.2a* and *MYB51* were also elevated to a greater extent in *RK3-ΔK* overexpression plants compared to those in WT plants (Figure 5E and Figure S8E). These data demonstrate that the intracellular kinase domain of RK3 is dispensable for its immune-potentiating function, indicating that *RK3* likely promotes flg22-triggered immunity through a non-catalytic mechanism. Consistently, RK3-ΔK maintained its constitutive interaction with FLS2 and BAK1 (Figure S9), similar to the full-length RK3. Together, these results demonstrate that the kinase domain of RK3 is not required for its positive regulatory role in flg22-triggered immunity. Instead, RK3 likely functions through its ligand-independent association with the receptor complex to modulate immune signaling.

### 3.5. Overexpression of RK3 in Arabidopsis and Tomato Enhanced Resistance to Pst DC3000

**Figure 6:**
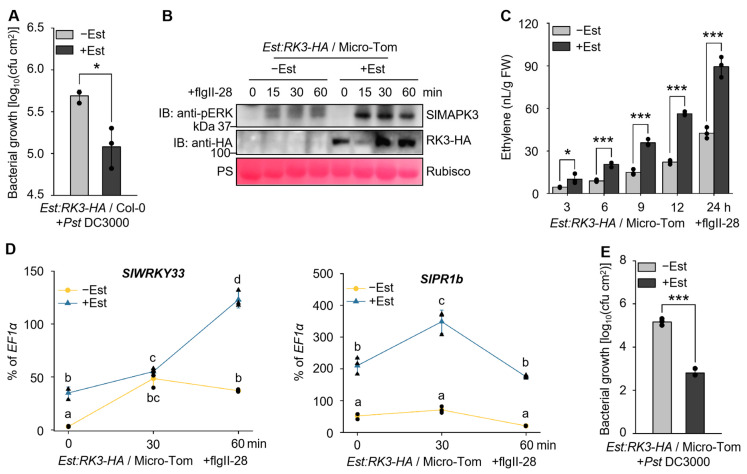
RK3 positively regulates PTI in both *Arabidopsis* and tomato. (**A**) Induced expression of RK3 enhances resistance to *Pst* DC3000 in *Arabidopsis*. Four-week-old rosette leaves of *Est:RK3-HA* transgenic plants were pre-treated with 10 µM estradiol (+Est) or solvent control (−Est) for 24 h, followed by infiltration with *Pst* DC3000 (OD_600_ = 0.0005). Bacterial titers were determined at 3 days post-inoculation (dpi). Data are means ± SD (*n* = 3 biological replicates). Student’s *t*-test: *, *p* < 0.05. (**B**–**E**) RK3 boosts immune outputs and confers bacterial resistance in tomato. *Est:RK3-HA* tomato plants were pre-treated with 10 µM estradiol (+Est) or solvent control (−Est) for 24 h before elicitation. (**B**) RK3 enhances flgII-28-induced phosphorylation of SlMAPK3. Total protein was extracted from 14-day-old seedlings treated with 300 nM flgII-28 and analyzed by immunoblotting. (**C**) RK3 potentiates flgII-28-triggered ethylene biosynthesis. Ethylene accumulation was measured by gas chromatography in 2.5-week-old seedlings exposed to 100 nM flgII-28. Data are means ± SD (*n* = 3, each replicate containing 3 seedlings). *, *p* < 0.05; ***, *p* < 0.001 (Student’s *t*-test). (**D**) RK3 upregulates flgII-28-induced defense gene expression. Transcript levels of *SlWRKY33* and *SlPR1b* were quantified by RT-qPCR in 2-week-old seedlings treated with 1 µM flgII-28, normalized to the reference gene *ACT*. Data are means ± SD (*n* = 3). Different lowercase letters indicate significant differences (two-way ANOVA with Tukey’s HSD post hoc test, *p* < 0.05). (**E**) RK3 confers enhanced resistance to *Pst* DC3000 infection in tomato. Four-week-old leaves were infiltrated with *Pst* DC3000 (OD_600_ = 0.0005). Bacterial populations were assessed at 3 dpi. Data are means ± SD (*n* = 3). ***, *p* < 0.001 (Student’s *t*-test). Black symbols in (**A**,**C**–**E**) represent individual data points.

Based on the established role of *RK3* in potentiating flg22-triggered immunity, we next assessed its capacity to enhance disease resistance in planta. Induced overexpression of *RK3* in transgenic *Arabidopsis* plants led to a significant reduction in bacterial growth following infection with *Pst* DC3000 (Figure 6A, Figures S10A and S12), demonstrating that *RK3* confers enhanced resistance to this bacterial pathogen in *Arabidopsis*. We next examined whether *RK3* overexpression confers broad-spectrum disease resistance. Compared to non-induced *Est:RK3-HA* plants (mock-treated), Est-induced *Est:RK3-HA* plants exhibited significantly enhanced resistance to the strain *Pst* DC3000-avrRpt2 (Figure S13), as well as to the necrotrophic fungal pathogen *B. cinerea* (Figure S14). Cross-family transfer of immune regulators represents a promising strategy for engineering disease resistance in crops [26,27,28]. The ability of RK3 to enhance immunity in *Arabidopsis* provides a foundation for exploring its utility in improving resistance in crop species.

To evaluate the potential of RK3 for improving disease resistance in crops, we generated the estradiol-inducible *RK3* transgenic tomato (*Solanum lycopersicum* cv. Micro-Tom), which exhibited no detectable effects on growth (Figure S11D). Induction of *RK3* expression in transgenic tomato plants strongly enhanced immune responses triggered by flgII-28, a tomato-perceived flagellin epitope [29]. This enhancement included potentiated activation of SlMAPK3 (Figure 6B and Figure S10B), elevated ethylene production (Figure 6C and Figure S10C), and increased expression of the defense-related genes *SlWRKY33* and *SlPR1b* (Figure 6D and Figure S10D). Consistent with this broad potentiation of PTI responses, ectopic expression of *RK3* in tomato plants markedly enhanced tomato resistance to *Pst* DC3000, with significantly decreased bacterial growth (Figure 6E and Figure S10E). These results demonstrate that *RK3* function is conserved between the crucifer and nightshade families, and suggest that it may be leveraged to engineer bacterial resistance in crops.

## 4. Discussion

LecRLKs constitute a large subfamily of RLKs, and their extracellular lectin domains are thought to reversibly bind carbohydrate ligands. Among LecRLKs, G-type members possess the most structurally complex extracellular structure, featuring multiple additional domains beyond the lectin domain. Notably, the SLG domain is homologous to S-locus glycoproteins that govern self- and interspecific incompatibility in *Brassica* species [30,31,32]. In self-compatible species such as *Arabidopsis*, S-locus-related genes have undergone functional divergence, enabling them to regulate vegetative growth and stress adaptation [15].

Many G-type LecRLKs are transcriptionally induced upon pathogen invasion, and accumulating evidence highlights their involvement in plant immunity. However, side-by-side comparison reveals that RK3 exhibits a unique combination of features that distinguish it from all previously characterized members. For instance, the identification of LORE as the receptor for the bacterial metabolite 3-OH-C10:0 underscores the importance of G-type LecRLKs in immune activation [18]. In contrast, RK3 does not directly bind a known ligand; instead, it constitutively associates with the FLS2-BAK1 complex to promote PTI. *RDA2* functions in [5-(3,4-dichlorophenyl)furan-2-yl]-piperidine-1-ylmethanethione (DFPM)-induced immune responses but does not participate in immune signaling mediated by peptide elicitors such as Pep1 and flg22 [33]; RK3, by contrast, positively regulates flg22-triggered immunity. In *Nicotiana benthamiana*, NbERK1 associates with BAK1 and SOBIR1 to regulate the perception of the expansin-like apoplastic elicitor protein, PcEXLX1 [34], thereby enhancing resistance to *Phytophthora capsici*. Although NbERK1 similarly interacts with BAK1, RK3 acts through a kinase-independent mechanism (as demonstrated by the functional RK3-ΔK variant), a feature not reported for NbERK1. In rice, *Pid2* confers resistance to *Magnaporthe oryzae* strain ZB15; intriguingly, a single amino acid substitution in the transmembrane domain distinguishes resistant and susceptible alleles, yet the *Pid2*-mediated signaling pathway remains largely unexplored [35]. Unlike *Pid2*, whose kinase domain is essential for resistance, *RK3* retains immune-enhancing activity even after deletion of its kinase domain. Conversely, *ERN1* negatively regulates immunity against root-knot nematodes, as evidenced by enhanced flg22-induced responses and disease resistance in *ern1* mutants, although the underlying mechanism remains unclear [36]. Thus, while *ERN1* acts as a negative regulator, *RK3* serves as a positive regulator of PTI, further highlighting their functional divergence. Despite the apparent importance of G-type LecRLKs in disease resistance, only a small fraction has been functionally characterized. Taken together, these comparisons indicate that RK3 represents a previously unrecognized mode of action among G-type LecRLKs—one that is distinct from the conventional roles of direct ligand receptors (LORE), negative regulators (ERN1), or signaling adaptors that strictly require kinase activity (NbERK1, Pid2).

In this study, we found that *RK3* was highly induced by *Pst* DC3000, *Pst* DC3000 strains carrying AvrRpt2 or AvrRpm1, as well as the fungal pathogen *Botrytis cinerea*. Biochemical analysis further revealed that RK3 constitutively interacted with FLS2 and BAK1 in a ligand-independent manner. Overexpression of *RK3* markedly enhanced flg22-induced hallmark responses including ROS burst, MAPK activation, ethylene production and increased expression of marker genes; conversely, the loss-of-function T-DNA insertion mutants attenuated these responses. These results suggest that RK3, through its constitutive association with the FLS2-BAK1 complex, promotes flg22-induced immune responses. Notably, deletion of the KD of RK3 did not affect the interaction of RK3 with FLS2 or BAK1, nor did it impair its ability to enhance flg22-induced immune responses, indicating that the ECD of RK3 is likely the functional region responsible for RK3-mediated immune potentiation. *RK3* was previously proposed to be involved in the switch from self-incompatibility to self-fertility in *A. thaliana* [31]. Our findings uncovered a distinct mode of function for *RK3* in self-compatible *A. thaliana*, suggesting a functional evolutionary shift in S-locus genes.

*RK3* and its two close homologs, *RK1* and *RK2*, belong to clade VI of G-lectin LecRLKs (subclade A1b) [25]. Our study revealed that RK3 promotes PTI by constitutively associating with an LRR-RLK-type PRR complex, and this function extends beyond FLS2, as ectopic expression of *RK3* in tomato also enhances flgII-28-induced immune responses. Interestingly, the other two members of clade VI, SBP1 (At4g27300) and SBP2 (At4g27290) (subclade A1c), form LRR-RLP-type receptor complexes through similar ligand-independent interactions with multiple RLPs and the co-receptors BAK1 and SOBIR1. Although kinase activity is dispensable for SBP1/SBP2 function in promoting ligand-induced immunity, their interactions are mediated by the kinase domain rather than the extracellular domain. Moreover, the contribution of SBP1/SBP2 to RLP-mediated immunity is comparable to that of SOBIR1 [37]. Taken together with our findings, these observations underscore the critical role of clade VI of G-lectin LecRLKs in sustaining full PTI output. Although pathogen- and flg22-induced expression of *RK1* and *RK2* is relatively low, we examined flg22-induced ethylene production in *rk2* T-DNA insertion lines and found no significant reduction compared with wild-type plants. For *RK1*, the very low level of transcriptional induction precluded a detailed PTI analysis in this study. These results suggest that *RK3* is a major contributor to flg22-triggered immunity. Whether *RK1* and *RK2* contribute to LRR-RLK-mediated immunity or other biological processes warrants future investigation.

In *Arabidopsis*, several RLKs belonging to distinct classes have been shown to interact with FLS2 and/or BAK1 to modulate flg22-induced immune signaling. For instance, the malectin-type RLK, FER, scaffolds the FLS2-BAK1 complex in a flg22-dependent manner and promotes its formation [38]. This function is inhibited by RALF peptide perception and requires the KD of FER [39]. Similarly, the Malectin/LRR-RLK, IOS1, constitutively associates with FLS2 and BAK1, and enhances MAMP-induced complex formation [40]. In contrast, three LRR-RLKs—BIR2, NIK1 and QSK1—negatively regulate flg22-triggered immunity through constitutive or flg22-dependent interactions with BAK1 and/or FLS2 [41,42,43]. Our study identified a G-type LecRLK, RK3, as a new type of positive regulator that interacts with FLS2 and BAK1 independently of flg22 stimulation. However, unlike canonical scaffolds that promote ligand-induced receptor heterodimerization or stabilize the components of the complex, RK3 does not appear to enhance flg22-triggered FLS2-BAK1 association or protein abundance. RK3 may help organize the signaling-competent nanodomain at the plasma membrane, facilitating the efficient recruitment or activation of downstream signaling components upon PAMP perception [44]. Future studies are needed to elucidate the mechanistic basis of RK3-mediated immune potentiation. Moreover, understanding how diverse positive and negative regulators coordinately interact with FLS2/BAK1 in vivo across spatiotemporal dimensions will provide a more comprehensive view of plant PTI regulation.

## 5. Conclusions

In this study, we identified RK3, a G-type LecRLK, as a positive regulator of PTI in *Arabidopsis*. *RK3* expression is rapidly and strongly induced upon bacterial and fungal pathogen challenge. Through gain- and loss-of-function analyses, we demonstrated that RK3 potentiates flg22-triggered immune outputs, including MAPK activation, ROS burst, ethylene production, defense gene expression, and enhanced resistance to *Pst* DC3000. Mechanistically, RK3 constitutively associates with the FLS2-BAK1 receptor complex in a ligand-independent manner, yet it does not promote flg22-induced FLS2-BAK1 heteromerization. Remarkably, the kinase domain of RK3 is dispensable for its immune-enhancing activity, as a kinase-deleted variant (RK3-ΔK) retains both the interaction with FLS2/BAK1 and the ability to boost flg22-triggered immunity. This reveals a non-canonical, kinase-independent mode of action for a G-type LecRLK in PTI signaling. Furthermore, ectopic expression of RK3 in tomato enhances flgII-28-induced immune responses and bacterial resistance, indicating that RK3 function is conserved between the crucifer and nightshade families, highlighting its potential as a tool for engineering broad-spectrum disease resistance in crops. Collectively, our findings uncover a previously unrecognized regulatory mechanism in plant immunity, expanding the functional repertoire of G-type LecRLKs and providing a valuable target for crop improvement.

## Data Availability

Data supporting the conclusions of this work can be obtained from the corresponding author upon reasonable request.

## Supplementary Materials

The following supporting information can be downloaded at https://www.mdpi.com/article/10.3390/biology15110822/s1: Figure S1: Transcriptional response of *G-type LecRLK* genes to flg22 elicitation; Figure S2: *RK3* shows the strongest transcriptional induction in response to diverse pathogens; Figure S3: RK3 enhances flg22-elicited immune responses in *Arabidopsis*; Figure S4: Disruption of RK3 attenuates flg22-induced defense responses in *Arabidopsis*; Figure S5: Flg22-induced ethylene production in the *rk3* mutants is initially comparable to that in wild-type *Arabidopsis* but significantly reduced at later time points; Figure S6: Quantitative analysis of the co-IP results shown in Figure 4B; Figure S7: Domain architecture of RK3 and the kinase-deleted RK3-ΔK; Figure S8: The kinase-deleted RK3 variant (RK3-ΔK) retains the capacity to enhance flg22-triggered immunity; Figure S9: RK3-ΔK constitutively interacts with FLS2 and BAK1; Figure S10: RK3 contributes positively to PTI responses in both *Arabidopsis* and tomato; Figure S11: Growth phenotypes of *Est:RK3-HA* overexpression lines, *rk3* mutants, and *Est:RK3-ΔK-HA* transgenic lines in *Arabidopsis* and tomato; Figure S12: Ethylene accumulation and camalexin content in *RK3*-overexpressing *Arabidopsis* upon inoculation with *Pst* DC3000; Figure S13: Resistance analysis of *RK3*-overexpressing *Arabidopsis* against *Pst* DC3000-avrRpt2; Figure S14: Resistance analysis of *RK3*-overexpressing *Arabidopsis* against *B. cinerea*; Table S1: Primers used for RT-qPCR in this study; Table S2: Primers used for cloning in this study.
