# Supplementary material for: RK3, a G-Type LecRLK, Interacts with FLS2 and BAK1 to Promote flg22-Triggered Immunity
Source: Biology (Basel). 2026 May 23;15(11):822. doi: 10.3390/biology15110822 (PMC13255945; doi:10.3390/biology15110822)
Supplement: Supplementary file 1 [file biology-15-00822-s001.zip › biology-4313614-supplementary.pdf]

**Supplementary Information (SI)**

# RK3, a G-type LecRLK, interacts with FLS2 and BAK1 to promote flg22-triggered immunity

**Authors:** Lu Zhang[1,2], Zhengdong Yuan[2], Lingya Yao[2], Hui Xiao[2,*]

**Affiliations:**

[1] School of Environmental and Chemical Engineering, Shanghai University, Shanghai 200444, China.

[2] Shanghai Key Laboratory of Plant Molecular Sciences, College of Life Sciences, Shanghai Normal University, Shanghai 200234, China.

* Correspondence: xiaohui@shnu.edu.cn

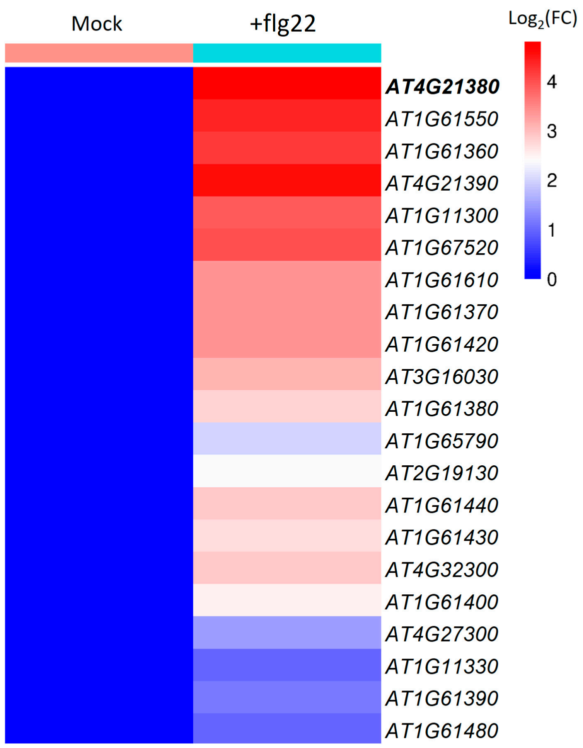


**Figure S1.** Transcriptional response of *G-type LecRLK* genes to flg22 elicitation. The heatmap shows significantly upregulated *Arabidopsis G-type LecRLK* genes at 90 minutes after flg22 treatment ($p < 0.05$). Data were obtained from the Expression Atlas (E-MTAB-9694).

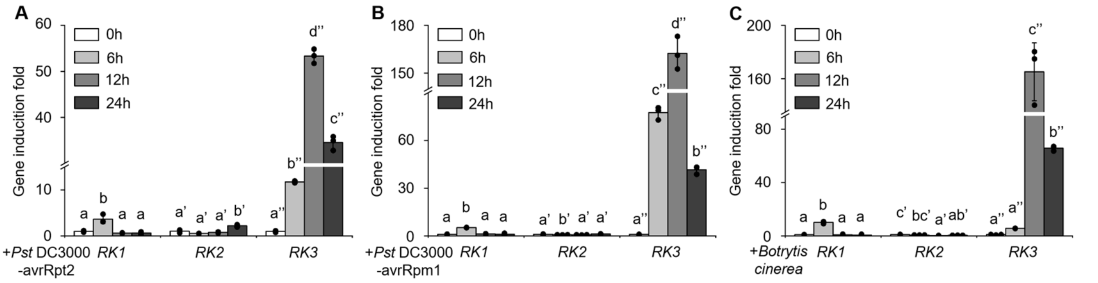


**Figure S2.** *RK3* shows the strongest transcriptional induction in response to diverse pathogens. Relative transcript levels of *RK1*, *RK2* and *RK3* in Col-0 seedlings were determined via RT-qPCR after inoculation with: **(A)** *Pst* DC3000-avrRpt2 ($OD_{600}$ = 0.02); **(B)** *Pst* DC3000-avrRpm1 ($OD_{600}$ = 0.02); or **(C)** the fungal pathogen *Botrytis cinerea* ($4 \times 10^6$ spores/mL). Values represent means ± SD of three biological replicates; black dots indicate individual data points. *EF1α* was employed as an internal control for normalization. Statistically significant differences across time points for each gene are indicated by different lowercase letters (one-way ANOVA with Tukey's HSD post-hoc test, $p < 0.05$).

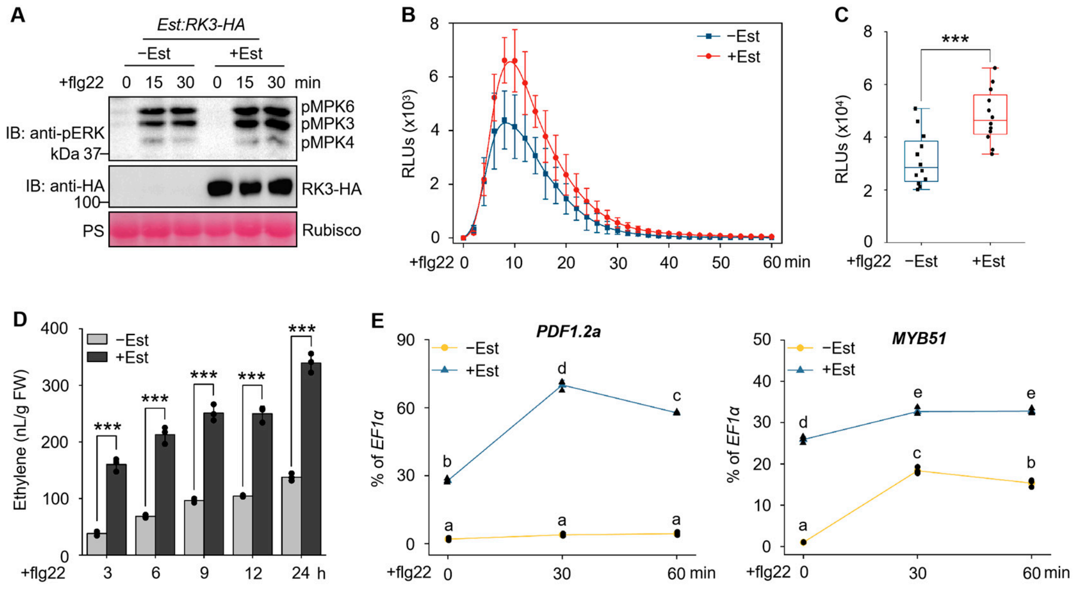


**Figure S3.** RK3 enhances flg22-elicited immune responses in *Arabidopsis*. *Est:RK3-HA* transgenic seedlings received a 24-h pre-treatment with 10 μM estradiol (+Est) or solvent control (–Est) before being challenged with 100 nM flg22. Plant tissues were obtained from axenically grown seedlings (10-day-old for panels **A** and **E**; 14-day-old for panel **D**) and soil-grown rosette leaves (4-week-old for panels **B** and **C**). **(A)** RK3 promotes flg22-induced MAPK phosphorylation. Immunoblotting was performed using anti-pERK (recognizing phosphorylated MPK3/6) and anti-HA (detecting RK3) antibodies. Rubisco staining was used as a loading control. **(B, C)** RK3 potentiates the flg22-triggered oxidative burst. ROS production was measured kinetically with a luminol-based assay. **(B)** Kinetic curves show mean relative light units (RLU) ± SD ($n = 12$ leaf discs). **(C)** Integrated ROS levels (total photon counts from 0 to 60 min) are presented as a box plot. Statistical significance was determined by Student's $t$-test (***, $p < 0.001$; $n = 12$). **(D)** RK3 augments flg22-induced ethylene emission. Ethylene accumulation was quantified by gas chromatography. Data are mean ± SD from three independent biological replicates (10 seedlings each). FW, fresh weight. ***, $p < 0.001$ (Student's $t$-test). **(E)** RK3 up-regulates defense-related gene expression following flg22 treatment. Transcript levels of *PDF1.2a* and *MYB51* were quantified via RT-qPCR and normalized to the internal reference *EF1α*. Data represent mean ± SD ($n = 3$). Distinct lowercase letters above bars indicate significant differences (two-way ANOVA with Tukey's HSD post-hoc test, $p < 0.05$). Black symbols in **(C–E)** represent individual data points.

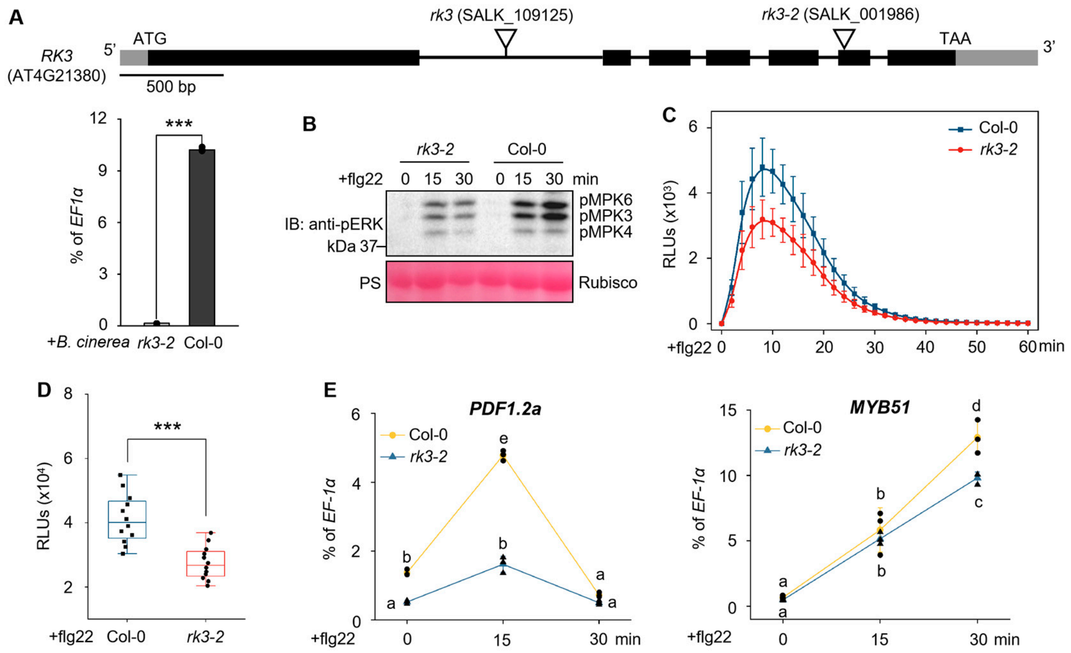


**Figure S4.** Disruption of RK3 attenuates flg22-induced defense responses in *Arabidopsis*. **(A)** Gene structure of *RK3* and transcriptional analysis of T-DNA insertion mutant. Schematic representation of the *RK3* genomic locus: black boxes indicate CDS, black lines represent introns, and gray boxes denote untranslated regions (UTRs). Insertion sites of *rk3* (SALK_109125) and *rk3-2* (SALK_001986) are marked. For transcriptional confirmation of *rk3-2* (which exhibits very low basal expression), seedlings were treated with *B. cinerea* to induce *RK3* expression. Total RNA was isolated from 10-day-old seedlings, and *RK3* mRNA levels were quantified by RT-qPCR using *EF1α* as the internal standard. Data represent mean ± SD ($n = 3$). ***, $p < 0.001$ (Student's $t$-test). **(B)** Reduced MAPK activation in the *rk3-2* mutant upon flg22 treatment. Seedlings were exposed to 100 nM flg22 for the indicated durations. Phosphorylated MPK3/4/6 were visualized by immunoblotting with anti-pERK antibodies. Rubisco staining was used as a loading reference. **(C, D)** Suppressed oxidative burst in *rk3-2* leaf discs following flg22 challenge. ROS generation was measured in real time using a luminol-based assay. **(C)** Kinetics of ROS release. Data are mean relative light units (RLU) ± SD ($n = 12$ leaf discs). RLU, relative light units. **(D)** Integrated ROS levels (total photon counts accumulated from 0 to 60 min) are displayed as a box plot ($n = 12$). Statistical significance was evaluated by Student's $t$-test (***, $p < 0.001$). **(E)** Diminished induction of defense marker genes in the *rk3-2* mutant after flg22 elicitation. Transcript abundances of *PDF1.2a* and *MYB51* were measured by RT-qPCR in 10-day-old seedlings treated with 100 nM flg22 for the indicated hours. Expression values were normalized to *EF1α*. Data are mean ± SD ($n = 3$ biological replicates). Different lowercase letters above bars denote statistically significant differences (two-way ANOVA followed by Tukey's HSD post-hoc test, $p < 0.05$). Black symbols in **(A)**, **(D)**, and **(E)** represent individual data points.

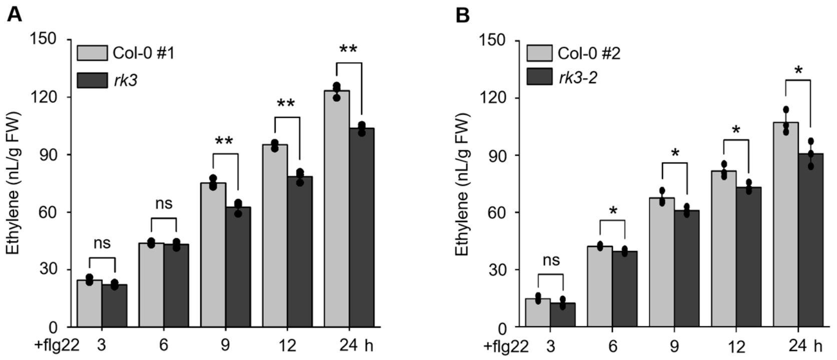


**Figure S5.** Flg22-induced ethylene production in the *rk3* mutants is initially comparable to that in wild-type *Arabidopsis* but significantly reduced at later time points. Ethylene accumulation was measured by gas chromatography in 2-week-old seedlings of the two *rk3* mutants and wild-type (Col-0) at the indicated hours after treatment with 100 nM flg22. Values are means ± SD ($n$ = 3 biological replicates); black dots indicate individual data points. Statistical significance at each time point was determined by Student's $t$-test (ns, not significant; ** $p < 0.01$; * $p < 0.05$). FW, fresh weight.

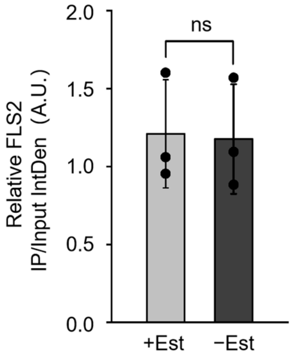


**Figure S6.** Quantitative analysis of the co-IP results shown in **Figure 4B**. The bar graph presents the relative protein level of FLS2, calculated as follows: the integrated density (IntDen, in arbitrary units, A.U.) of the FLS2 band in the immunoprecipitation (IP) and input samples was measured using ImageJ software. The FLS2 protein level was then normalized by the ratio of IP to Input (IP/Input). The horizontal axis indicates the two treatment conditions (+Est and −Est) as described for the main figure **(Figure 4B)**. Data are derived from three independent replicates, expressed as mean ± SD, with individual data points shown as black dots. Statistical analysis was performed using Student's *t*-test, and no significant difference was detected between the two conditions.

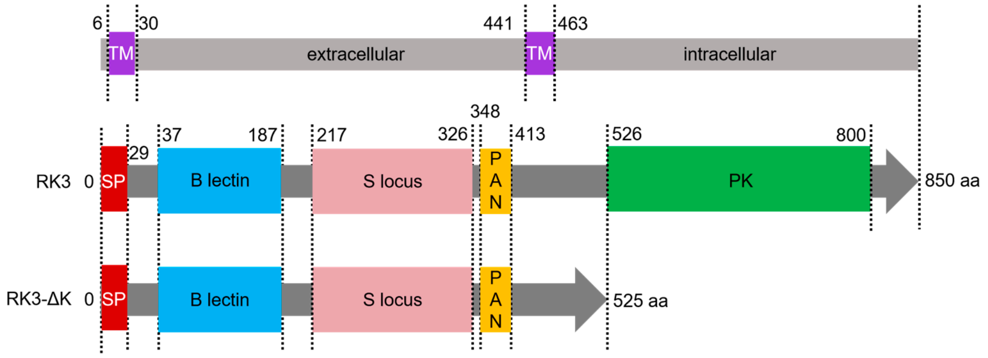


**Figure S7.** Domain architecture of RK3 and the kinase-deleted RK3-ΔK. The schematic shows the predicted domains (SP, B-lectin, S-locus, PAN, TM, and PK) of RK3 and RK3-ΔK, as identified by SMART and DeepTMHMM-1.0, and indicates the extracellular, transmembrane, and intracellular regions. Abbreviations: SP, signal peptide; S locus, S-locus glycoprotein domain; PAN, plasminogen-apple-nematode domain; PK, protein kinase domain; TM, transmembrane domain.

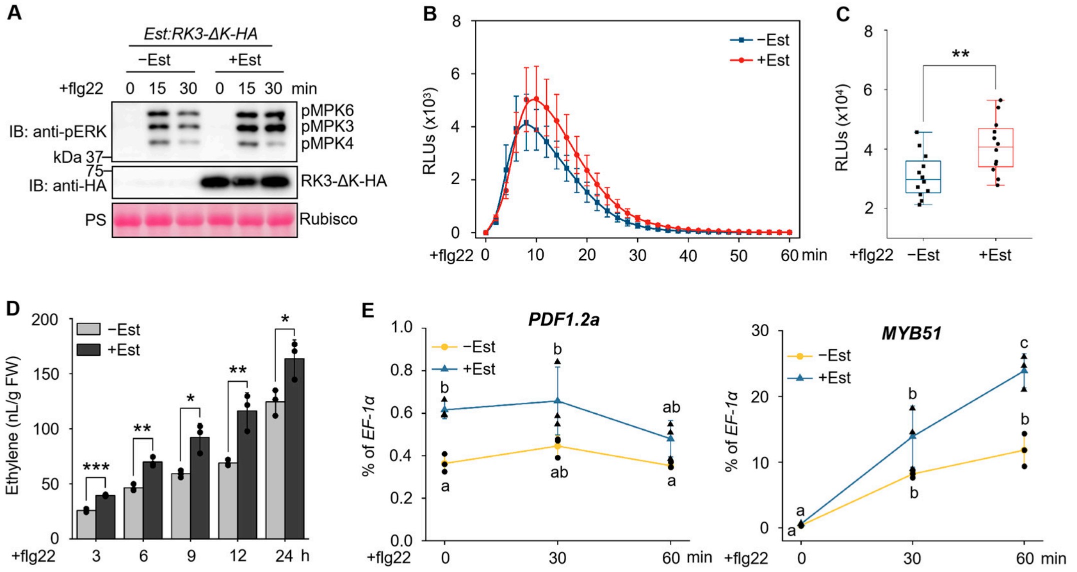


**Figure S8.** The kinase-deleted RK3 variant (RK3-ΔK) retains the capacity to enhance flg22-triggered immunity. *Est:RK3-ΔK-HA* transgenic seedlings received a 24-h pre-treatment with 10 µM estradiol (+Est) or solvent control (–Est) before being challenged with 100 nM flg22. Plant materials were derived from aseptically grown seedlings (10-day-old for **A**,**E**; 14-day-old for **D**) or soil-grown rosette leaves (4-week-old for **B**,**C**). **(A)** RK3-ΔK expression promotes flg22-induced MAPK phosphorylation. Immunoblots using anti-pERK (recognizing MPK3/4/6) and anti-HA (detecting RK3-ΔK) are shown. Rubisco staining was used as a loading control. **(B, C)** RK3-ΔK potentiates the flg22-triggered oxidative burst. ROS production was monitored over 60 min following flg22 application. **(B)** Kinetic curves depict mean relative luminescence units (RLU) ± SD ($n = 12$ leaf discs). **(C)** Integrated ROS levels (total photon counts from 0 to 60 min) are presented as a box plot. Statistical significance was determined by Student's *t*-test (**, $p < 0.01$; $n = 12$). **(D)** RK3-ΔK augments flg22-induced ethylene emission. Ethylene accumulation was quantified by gas chromatography in 14-day-old seedlings at the indicated time points after elicitation. Data represent mean ± SD ($n = 3$). Student's *t*-test: *, $p < 0.05$; **, $p < 0.01$; ***, $p < 0.001$. **(E)** RK3-ΔK up-regulates defense marker gene expression upon flg22 treatment. Transcript levels of *PDF1.2a* and *MYB51* were measured by RT-qPCR in 10-day-old seedlings subjected to 100 nM flg22 for the indicated hours. Expression values were normalized to *EF1α*. Data are mean ± SD ($n = 3$ biological replicates). Different lowercase letters above bars indicate significant differences (two-way ANOVA with Tukey's HSD post-hoc test, $p < 0.05$). Black symbols in **(C–E)** represent individual data points.

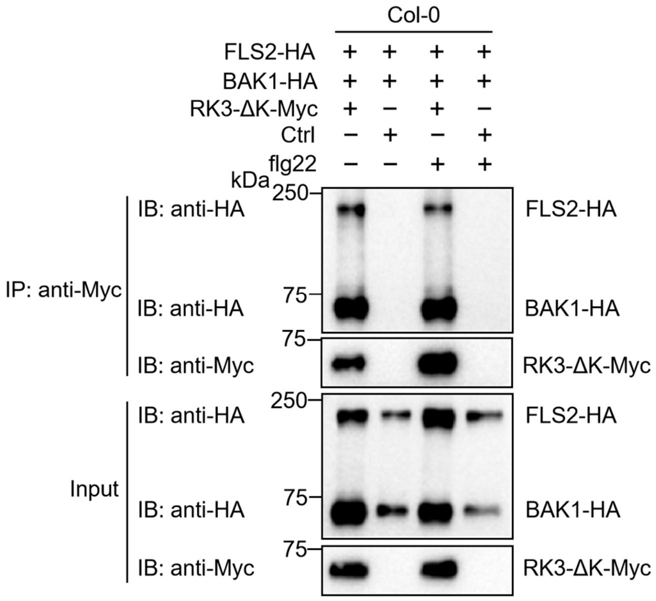


**Figure S9.** RK3-ΔK constitutively interacts with FLS2 and BAK1. RK3-ΔK-Myc was co-expressed with FLS2-HA or BAK1-HA in protoplasts treated with 100 nM flg22 or mock for 10 min. Protein complexes were immunoprecipitated with anti-Myc beads and analyzed by immunoblotting with anti-HA and anti-Myc antibodies. Ctrl, vector control. "+" and "–" indicate the presence or absence of the indicated component (flg22, or maxi-prep DNA).

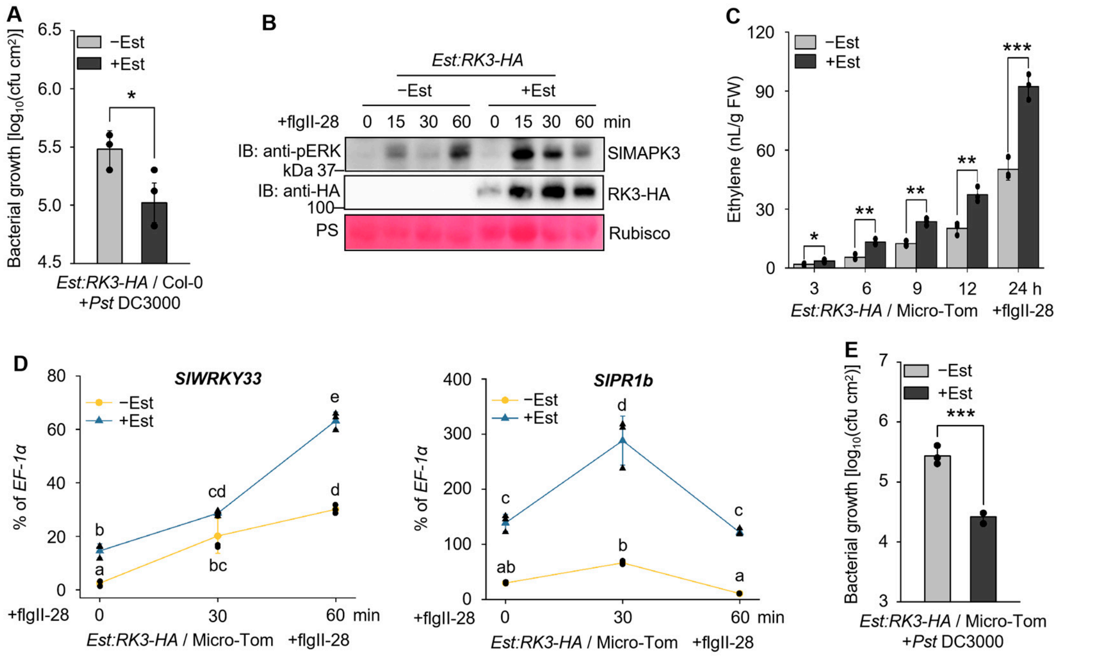


**Figure S10.** RK3 contributes positively to PTI responses in both *Arabidopsis* and tomato. **(A)** Induced RK3 expression confers enhanced resistance to *Pst* DC3000 in *Arabidopsis*. Rosette leaves of 4-week-old *Est:RK3-HA* transgenic plants were pre-treated with 10 µM estradiol (+Est) or solvent control (–Est) for 24 h, then infiltrated with *Pst* DC3000 ($OD_{600}$ = 0.0005). Bacterial loads were measured at 3 days post-inoculation (dpi). Data are means ± SD ($n = 3$ biological replicates). Student's *t*-test: *, $p < 0.05$. **(B–E)** RK3 potentiates immune signaling and bacterial resistance in tomato. *Est:RK3-HA* tomato plants were pre-treated with 10 µM estradiol (+Est) or solvent control (–Est) for 24 h prior to elicitation. **(B)** RK3 enhances flgII-28-induced SlMAPK3 phosphorylation. Total protein was extracted from 14-day-old seedlings treated with 300 nM flgII-28 and analyzed by immunoblotting. **(C)** RK3 amplifies flgII-28-triggered ethylene production. Ethylene accumulation was quantified by gas chromatography in 2.5-week-old seedlings exposed to 100 nM flgII-28. Values are means ± SD ($n = 3$, each replicate containing 3 seedlings). *, $p < 0.05$; **, $p < 0.01$; ***, $p < 0.001$ (Student's *t*-test). **(D)** RK3 up-regulates flgII-28-induced defense marker transcripts. Transcript abundances of *SlWRKY33* and *SlPR1b* were measured by RT-qPCR in 2-week-old seedlings treated with 1 µM flgII-28 and normalized to the reference gene *ACT*. Data are means ± SD ($n = 3$). Different lowercase letters denote significant differences (two-way ANOVA with Tukey's HSD post-hoc test, $p < 0.05$). **(E)** RK3 confers increased resistance to *Pst* DC3000 infection in tomato. Four-week-old leaves were infiltrated with *Pst* DC3000 ($OD_{600}$ = 0.0005). Bacterial populations were assessed at 3 dpi. Data are means ± SD ($n = 3$, individual points shown). ***, $p < 0.001$ (Student's *t*-test). Black symbols in **(A, C, D, E)** represent individual data points.

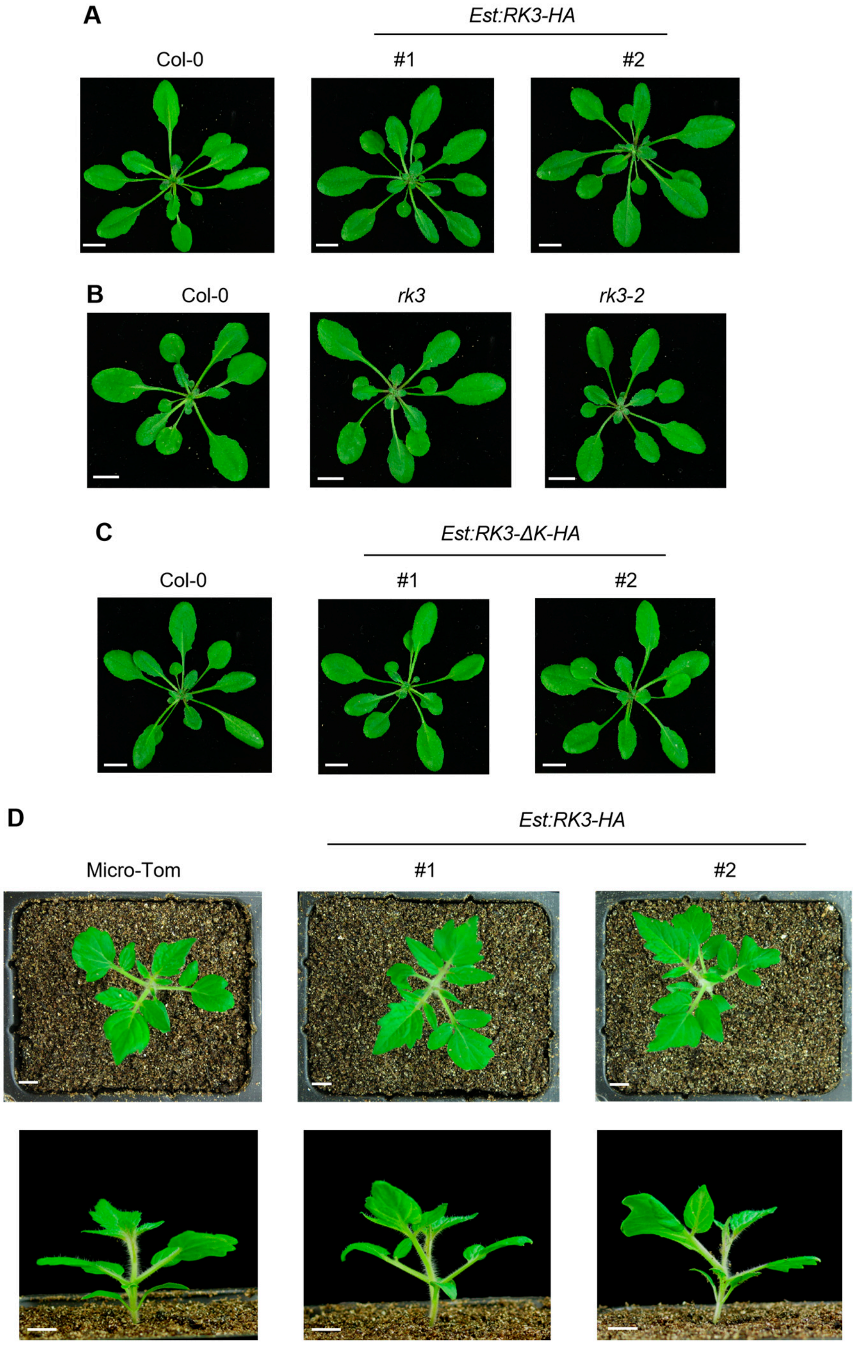


**Figure S11.** Growth phenotypes of *Est:RK3-HA* overexpression lines, *rk3* mutants, and *Est:RK3-ΔK-HA* transgenic lines in *Arabidopsis* and tomato. **(A)** Phenotypes of *Est:RK3-HA* overexpressing *Arabidopsis* plants. Four-week-old Col-0 wild-type and two independent transgenic lines (#1 and #2) were grown under identical conditions. Scale bar = 1 cm. **(B)** Rosette leaf morphology of the *rk3* mutants. Comparison between 4-week-old wild-type (Col-0) and *rk3* mutants plants. Scale bar = 1 cm. **(C)** Growth phenotypes of *Est:RK3-ΔK-HA* transgenic *Arabidopsis* lines. Col-0 and two independent lines (#1 and #2) were grown in soil for 4 weeks under the same conditions. Scale bar = 1 cm. **(D)** Growth phenotypes of *Est:RK3-HA* transgenic

tomato plants. Four-week-old transgenic lines and wild-type Micro-Tom were grown under identical conditions. Scale bar = 2 cm.

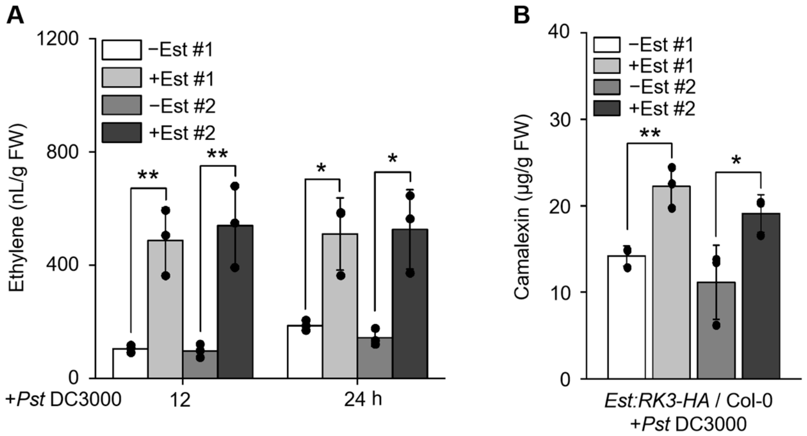


**Figure S12.** Ethylene accumulation and camalexin content in *RK3*-overexpressing *Arabidopsis* upon inoculation with *Pst* DC3000. **(A)** Ethylene accumulation assay. T1 seedlings were inoculated with *Pst* DC3000 ($OD_{600}$ = 0.02). Ethylene production was measured by gas chromatography at 12 h and 24 h post-inoculation. Three biological replicates were performed, each consisting of 10 seedlings. Data are presented as mean ± SD ($n$ = 3); FW, fresh weight. Statistical analysis was performed using Student's $t$-test; * and ** indicate $p < 0.05$ and $p < 0.01$. **(B)** Camalexin content assay. The same T1 material was used. At 24 h post-inoculation, camalexin accumulation in leaves was determined using a microplate reader. Briefly, after weighing the samples used for ethylene measurement, 200 μL of supernatant was transferred to a black 96-well microplate, along with a series of camalexin standards and a blank control (SW solution). Fluorescence was measured at excitation 310 nm and emission 390 nm. Camalexin content was calculated based on a standard curve. Data are shown as mean ± SD ($n$ = 3). *, $p < 0.05$; **, $p < 0.01$ (Student's $t$-test). Black dots in **(A, B)** represent individual data points.

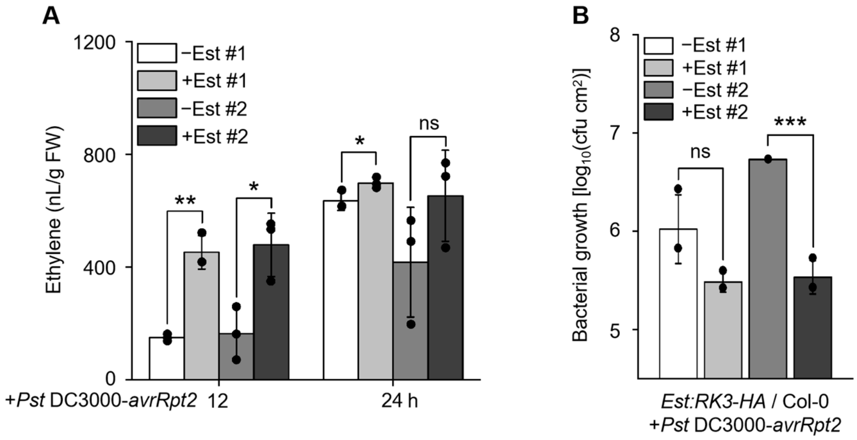


**Figure S13.** Resistance analysis of *RK3*-overexpressing *Arabidopsis* against *Pst* DC3000-avrRpt2. **(A)** Ethylene accumulation. T1 seedlings were inoculated with *Pst* DC3000-avrRpt2 ($OD_{600}$ = 0.02). Ethylene production was measured by gas chromatography at the indicated time points. Data are presented as mean ± SD ($n$ = 3, each replicate containing 10 seedlings). *, $p < 0.05$; **, $p < 0.01$; ns, not significant (Student's $t$-test). **(B)** Bacterial growth assay. Four-week-old T2 plants were inoculated with *Pst* DC3000-avrRpt2 ($OD_{600}$ = 0.0005). Bacterial titers in leaves were determined at 3 days post-inoculation. Data are shown as mean ± SD ($n$ = 3). Statistical analysis was performed using Student's $t$-test. ***, $p$ < 0.001; ns, not significant. Black dots in **(A, B)** represent individual data points.

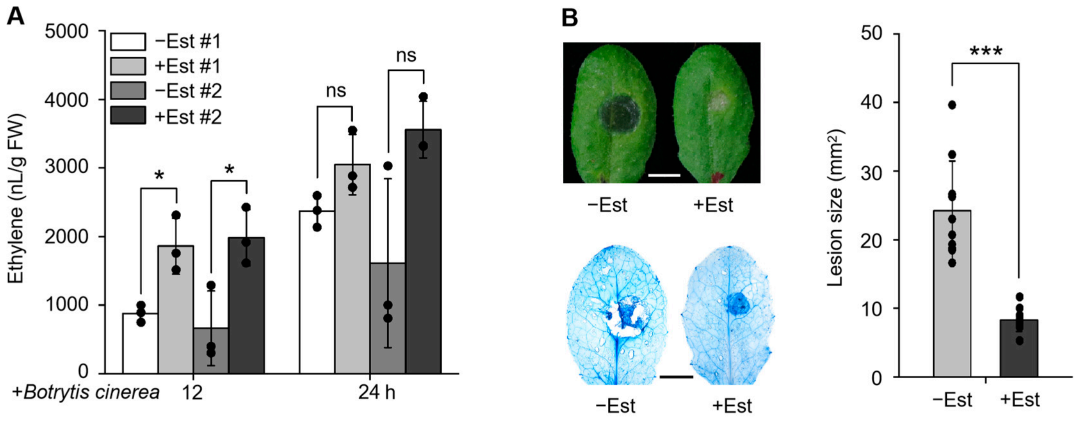


**Figure S14.** Resistance analysis of *RK3*-overexpressing *Arabidopsis* against *B. cinerea.* **(A)** Ethylene measurement. Two-week-old T1 *Est:RK3-HA* transgenic seedlings were inoculated with *B. cinerea* at a concentration of $4 \times 10^6$ spores/mL. Ethylene production was measured at the indicated time points. Data are presented as mean ± SD ($n$ = 3 biological replicates, each replicate consisting of 10 seedlings). Statistical analysis was performed using Student's $t$-test; * indicates $p < 0.05$, and ns indicates not significant ($p \geq 0.05$). **(B)** Disease resistance assay. Rosette leaves of four-week-old T2 plants were inoculated with *B. cinerea* spore suspension ($2 \times 10^5$ spores/mL). Lesion phenotypes were observed at 2 days post-inoculation. Fungal hyphal expansion was visualized by trypan blue staining. Lesion areas were quantified using ImageJ software ($n$ = 8). *** indicates $p < 0.001$ (Student's $t$-test). Scale bar = 0.5 cm. Black dots in **(A, B)** represent individual data points.

**Table. S1** Primers used for RT-qPCR in this study.

| **Primer Name** | **Sequence** |
|---|---|
| *RK1-RT-F* | AGGTGGATTCGGAATTGTTTAC |
| *RK1-RT-R* | CCTTGTGAGGACATTTTCGATAG |
| *RK2-RT-F* | CCGTGATCTTAATCTCCTTGGT |
| *RK2-RT-R* | GAATTCTGATGACAAAGCATCG |
| *RK3-RT-F* | ATTCTACAATTCGGACCGTGAC |
| *RK3-RT-R* | CATGTTGCCTGAATGTTGATG |
| *EF1α-RT-F* | TGAGCACGCTCTTCTTGCTTTCA |
| *EF1α-RT-R* | GGTGGTGGCATCCATCTTGTTACA |
| *PDF1.2a-RT-F* | CCTTATCTTCGCTGCTCTTGTT |
| *PDF1.2a-RT-R* | ATGTTTGGCTCCTTCAAGGTTA |
| *MYB51-RT-F* | AAAAGCTGGACTCAAGAGATGC |
| *MYB51-RT-R* | AGTCCACGAGCTATAGCAGACC |
| *SlPR1b-RT-F* | ATTCATTCTGGTGCTGGGGA |
| *SlPR1b-RT-R* | GTTGCGCCAGACTACTTGAG |
| *SlWRKY33-RT-F* | GAGATGGAAGGGTGACAATGAA |
| *SlWRKY33-RT-R* | GGTTCGGATTTCCTTTGACAA |
| *ACT-RT-F* | TGAGCTTCGAGTTGCTCCTGA |
| *ACT-RT-R* | AGCACAGCCTGGATAGCAACA |

**Table. S2** Primers used for cloning in this study.

| **Primer Name** | **Sequence** |
| --- | --- |
| *RK3-F-SalI-BamHI* | ACGCGTCGACGGATCCATGAGAGGTTTACCAAATTTCTACC |
| *RK3-R-SpeI-StuI* | GGACTAGTAGGCCTCCGAGCATCAAGGACCG |
| *RK3-ΔK-R-SpeI-StuI* | GGACTAGTAGGCCTATTGTTTGTGGCCATGGCA |
| *FLS2-F-SalI-BamHI* | ACGCGTCGACGGATCCATGAAGTTACTCTCAAAGACCTTTTTGAT |
| *FLS2-R-SpeI-SmaI* | GGACTAGTCCCGGGAACTTCTCGATCCTCGTTACGATC |
| *BAK1-F-XhoI-BamHI* | CCGCTCGAGGGATCCTGGAACGAAGATTAATGATCCCTT |
| *BAK1-R-XbaI-StuI* | GCTCTAGAAGGCCTTCTTGGACCCGAGGGGTATT |
